# Temporal stability of Bayesian belief updating in perceptual decision-making

**DOI:** 10.3758/s13428-023-02306-y

**Published:** 2023-12-21

**Authors:** Isabella Goodwin, Robert Hester, Marta I. Garrido

**Affiliations:** 1 Melbourne School of Psychological Sciences, The University of Melbourne, Parkville Campus, Melbourne, Victoria 3010 Australia; 2 Graeme Clark Institute for Biomedical Engineering, The University of Melbourne, Melbourne, Victoria Australia

**Keywords:** Individual differences, Perceptual decision-making, Reliability, Bayesian belief updating

## Abstract

Bayesian inference suggests that perception is inferred from a weighted integration of prior contextual beliefs with current sensory evidence (likelihood) about the world around us. The perceived precision or uncertainty associated with prior and likelihood information is used to guide perceptual decision-making, such that more weight is placed on the source of information with greater precision. This provides a framework for understanding a spectrum of clinical transdiagnostic symptoms associated with aberrant perception, as well as individual differences in the general population. While behavioral paradigms are commonly used to characterize individual differences in perception as a stable characteristic, measurement reliability in these behavioral tasks is rarely assessed. To remedy this gap, we empirically evaluate the reliability of a perceptual decision-making task that quantifies individual differences in Bayesian belief updating in terms of the relative precision weighting afforded to prior and likelihood information (i.e., sensory weight). We analyzed data from participants (*n* = 37) who performed this task twice. We found that the precision afforded to prior and likelihood information showed high internal consistency and good test–retest reliability (ICC = 0.73, 95% CI [0.53, 0.85]) when averaged across participants, as well as at the individual level using hierarchical modeling. Our results provide support for the assumption that Bayesian belief updating operates as a stable characteristic in perceptual decision-making. We discuss the utility and applicability of reliable perceptual decision-making paradigms as a measure of individual differences in the general population, as well as a diagnostic tool in psychiatric research.

**Supplementary Information:**

The online version contains supplementary material available at 10.3758/s13428-023-02306-y.

## Introduction

Bayesian accounts of perception suggest that the brain creates an internal model of the world to infer the cause of sensory input. This inference is generated via a weighted combination of prior expectations and incoming sensory observations which are used to estimate the state of any given environment (Hohwy, 2013, 2020; Knill & Pouget, 2004). Intrinsic uncertainty associated with prior and sensory (or likelihood) information determines how they are relatively weighted to form an internal representation of the world. Accurately incorporating this information into a veridical model of one’s environment is essential for optimizing perception and effective perceptual decision-making (Friston, 2005, 2008). This framework, grounded in predictive processing, is useful for understanding complex mechanisms underlying healthy information processing. In turn, it also aids a phenomenological explanation for how aberrancies in the precision afforded to different types of information may characterize altered perceptual experiences (Hohwy, 2020). While these aberrancies in belief updating are thought to underlie symptoms of psychopathology and trait-like correlates in non-clinical populations (Fromm et al., 2023; Gibbs-Dean et al., 2023; Karvelis et al., 2023), the assumption that individual differences in belief updating is a stable characteristic is yet to be verified. This can offer valuable insight into the cognitive mechanisms underpinning behavior (Tulver et al., 2019).

Disruptions in the precision weighting afforded to prior and likelihood information have been used as a framework for understanding a spectrum of clinical disorders including psychosis and schizophrenia (Adams et al., 2013; Sterzer et al., 2018), autism spectrum disorder (Palmer et al., 2015, 2017; Randeniya et al., 2021), mood disorders (Kraus et al., 2021; Putica et al., 2022) and more. This framework has also been used to investigate individual differences in non-clinical populations such as autistic traits, schizotypy, and trait anxiety (Goodwin et al., 2022; Kraus et al., 2021; Kreis et al., 2023). These individual differences in perceptual inference can be conceptualized across a continuum from stable characteristics in the general population, to more severe aberrancies in clinical disorders. This is particularly important in understanding the development and trajectory of disorders, integrating a transdiagnostic approach into understanding symptomatology (Gibbs-Dean et al., 2023; Lyndon & Corlett, 2020).

Investigating symptomatology and analogous sub-clinical characteristics of such disorders relies on the assumption that distinct perceptual phenotypes result from differences in the precision weighting of prior beliefs and sensory evidence (van Leeuwen et al., 2021). This assumes that perceptual differences in belief updating remain stable across individuals over time. While this offers important empirical utility in understanding of symptom formation, the assumption that Bayesian information integration is a stable characteristic has received little attention thus far. Furthermore, research that empirically verifies the reliability of behavioral measures to assess cognitive performance is scarce. This could largely impact the interpretation and application of such measures in our understanding of individual differences in cognitive function and its usefulness for clinical translation (Parsons et al., 2019). Therefore, it is important to verify whether measures with high variance between individuals (such as the precision weighting afforded to prior and likelihood information) can be attributed to stable individual differences in cognitive mechanisms (Parsons et al., 2019; Rouder et al., 2019).

Several studies have investigated the temporal stability of behavioral tasks that are used to measure stable characteristics in other measures of cognitive performance. For example, this has been assessed in model-based correlates of compulsivity (Brown et al., 2020) and processes underlying self-regulation (Zech et al., 2022). Behavioral and computational measures of a probabilistic reversal learning task demonstrated high reliability (Waltmann et al., 2022), validating its interpretability of individual differences in cognitive flexibility, as well as deviances across psychiatric populations in a transdiagnostic manner. This recent exploration into the psychometrics of cognitive behavioral measures have coincided with a shift in methodology to focus on hierarchical modeling that includes trial-level variation across a task, rather than traditional approaches that focus on average scores. This novel approach allows reliability estimates to vary at the individual level, meaning that researchers can directly test for homogenous within-person variance of behavioral measures (Williams et al., 2022).

Of relevance to the predictive coding framework, a recent study investigated the test–retest reliability in individuals’ cross modal usage of priors in the perception of bistable visual stimuli (Pálffy et al., 2021). This paradigm adjudicated between individuals’ reliance on auditory versus visual associative cues in visual perception. Importantly, substantial inter-individual variability suggested large differences in the relative use of acoustic compared to visual prior information, which researchers found to be temporally stable over two testing sessions. Despite this, it is unclear whether other aberrancies in Bayesian perceptual belief updating act as a temporally stable characteristic, particularly when the uncertainty associated with both prior *and* likelihood information are differentially altered.

This study aims to investigate whether a general reliance on likelihood relative to prior information can be considered a stable characteristic. This will be investigated with a behavioral paradigm that orthogonally manipulates prior and likelihood information and is known to yield systematic variation between individuals (Vilares et al., 2012). Along with traditional test–retest reliability measures of task performance, we will also use a hierarchical modeling approach that accounts for trial-by-trial estimates of sensory weight. This approach has consistently been shown to produce better reliability estimates in reliability, as variance in trial-by-trial data is incorporated. The behavioral paradigm will also allow us to determine whether average metrics of subjective uncertainty associated with likelihood and prior information remains stable over two testing sessions. Note however, that trial-by-trial estimates of subjective likelihood variance and subjective prior variance are not available, as these parameters are only calculated as an average metric across conditions or across the whole task.

## Method

### Participants

We aimed to recruit at least 30 participants, based on the number of healthy participants recruited in previous coin task paradigms (Randeniya et al., 2021; Trapp & Vilares, 2020; Vilares et al., 2012; Vilares & Kording, 2017) and based on similar test–retest studies investigating individual differences in perceptual inference (Pálffy et al., 2021). To account for potential dropout and exclusion, we initially recruited 62 participants who completed the first testing session. The final sample consisting of 37 participants who completed both testing sessions (29 female, six male, two non-binary, age range = 18–56, *M* = 21.87, *SD* = 6.72). Participants completed the second testing session 12–17 days after the first session (*M* = 14.22, *SD* = 1.80), with the aim of averaging 2 weeks between testing sessions.

Participants were recruited through the University of Melbourne research experience program. Participants were at least 18 years of age and had corrected-to-normal vision. They were asked about their highest level of education, left or right handedness, whether English was their first language, how many years they had been speaking English if not, vision impairments, previous diagnosis of serious neurological conditions and/or emotional or psychological disorders, and any other conditions that might affect performance. All participants gave informed consent and received credit towards a university subject completion for participation. The study was approved by the University of Melbourne Human Research Ethics Committee (Ethics ID: 20592).

### Experimental design

#### Procedure

Participants were recruited through a university online system (SONAR) that allowed completion of both testing sessions online, on their own laptops. Participants initially provided demographic details via Qualtrics (www.qualtrics.com), and were then directed to Pavlovia (www.pavlovia.com) to complete the behavioral task. The paradigm that participants completed at the second timepoint was the same as the paradigm at the first timepoint.

#### Coin task

**Fig. 1:**
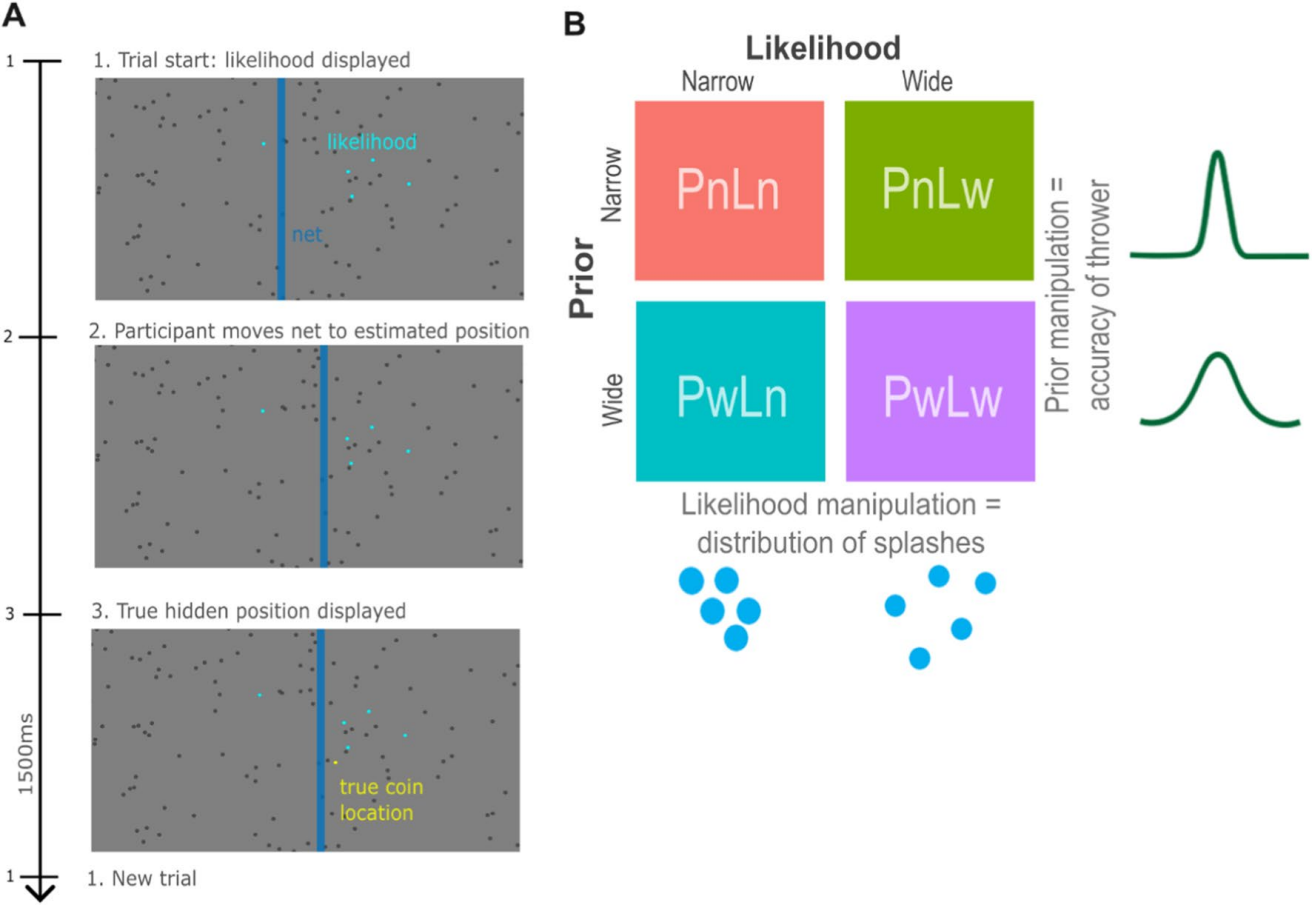
Coin task paradigm as adapted from Vilares et al. (2012) demonstrating **A** the time course of a single trial and **B** the task conditions. *Note.* A) Time course of a single exemplar trial: participants are shown five blue dots to represent splashes of the location of a coin being thrown into a pond. They are then asked to move the blue bar/net to where they estimate the coin’s location to be, after which the coin’s true location is revealed, and they move onto the next trial. B) Task design as adapted from Vilares et al. (2012): the four conditions of the task are visually depicted including two types of likelihood as manipulated through the distribution of splashes on each trial (Ln = narrow likelihood; Lw = wide likelihood) and two types of prior as manipulated through the accuracy of the thrower on each block (Pn = narrow prior; Pw = wide prior)

Participants performed a decision-making task where they were asked to guess the position of a hidden target on a screen, requiring them to integrate both noisy sensory evidence and prior expectation of the target’s location. More specifically, participants were told a coin was being thrown into a pond and were asked to guess where the coin had fallen. Likelihood and prior variance were manipulated with a two-by-two factorial design with narrow and wide variance, respectively. On each trial, five blue dots denoted “splashes” produced by the coin falling in. The variance of these splashes changed on each trial as an index of either narrow or wide likelihood conditions. The position of these splashes was drawn from a Gaussian distribution centered on the (hidden) location of the coin, with standard deviation of either 6% of the screen width (*SD* = 0.096; narrow likelihood trials) or 15% of the screen width (*SD* = 0.24; wide likelihood trials). An example trial is shown in Fig. 1. Participants were also informed that the person throwing the coin changed between blocks, and one thrower was more accurate than the other. They were told that both throwers aimed at the screen center (indicating the mean of the prior). Although they were not explicitly told which thrower was better or worse, this could be inferred through the distribution of previous coin locations from trial-to-trial. The location of the coin was drawn from a second, independent Gaussian distribution centered on the middle of the screen, with a standard deviation of either 2.5% of the screen width (*SD* = 0.04; narrow prior blocks) or 8.5% of the screen width (*SD* = 0.136; wide prior blocks). The four conditions are visually depicted in Fig. 1.

While the variance of the likelihood changed pseudorandomly from trial-to-trial (counterbalanced across all trials), the variance of the prior changed from block to block, with the order (thrower A vs thrower B) also counterbalanced across participants. Thus, there were four conditions: narrow prior and narrow likelihood (PnLn), narrow prior and wide likelihood (PnLw), wide prior and narrow likelihood (PwLn), and wide prior and wide likelihood (PwLw).

For each trial, participants were instructed to move a net (blue bar) horizontally across the screen to indicate where they thought the coin had landed. The true position of the coin (represented as a yellow dot) was then shown for 1500 ms. Scoring was tallied across each trial, with a point earned each time any part of the coin lay within the net. Participants were provided with two blocks of two practice trials before completing the main task. The main task consisted of two blocks per thrower, with each block containing 75 trials each (resulting in 300 trials total).

#### Likelihood only task

Prior to completing the coin task, participants completed the likelihood-only estimation task as a measure of subjective likelihood variance or sensory noise. The setup of this task was similar to the main task, without the incorporation of the prior condition. This provided an estimation of how participants perceived the center of the splashes on their own, without prior knowledge. Participants were asked to estimate where they thought the true coin location was, which was always the center of the displayed splashes, by moving the net horizontally across the screen. The true coin location (represented as a yellow dot) was revealed at the end of each trial, providing feedback on participants estimations. This task consisted of 100 trials, with an even number of wide and narrow likelihood distributions.

### Behavioral analysis

Successful performance of the task required participants to move the net to the most likely location of the hidden coin. Using Bayes rule, we can determine what the optimal estimate of the position of the coin would be on each trial (Körding & Wolpert, 2004; Vilares et al., 2012):

1 $${X}_{est}=\frac{\sigma_L^2}{\sigma_L^2+{\sigma}_P^2}\ {\mu}_P+\frac{\sigma_P^2}{\sigma_L^2+{\sigma}_P^2}\ {\mu}_L$$

where *X*_*est*_ is the estimated position of the coin (i.e., participants responses on each trial), (*μ*_*P*_, *μ*_*L*_) represent the prior and likelihood means and (${\sigma}_P^2,{\sigma}_L^2$) represent the prior and likelihood variances, respectively. In our experiment, the mean of the prior was kept constant (the center of the screen, *μ*_*P*_), while the mean of the likelihood was determined by the center of the five blue dots in each trial ( *μ*_*L*_).

#### Performance

Performance in the likelihood-only task was characterized by the average distance between participants’ estimates of the coin location (net location) and the true center of the splashes (i.e., mean estimation error). Similarly, performance in the coin task was characterized by the average distance between participants’ estimates (net location) and the true location of the coin.

#### Overall sensory weight (likelihood vs prior reliance)

To estimate participants reliance on likelihood relative to prior information, we fitted a linear regression to participants’ estimates of the coin’s position for each trial (*X*_*est*_) as a function of the center of the splashes (i.e., the likelihood mean, *μ*_*L*_):

2 $$sw=\frac{\sigma_P^2}{\sigma_L^2+{\sigma}_P^2}$$

where *sw* is the slope of the linear regression, which indicates how much each participant relies on likelihood information. A slope closer to 1 indicates a greater reliance on the likelihood information, while a slope closer to 0 indicates greater reliance on prior information. A slope between 0 and 1 indicates that participants integrate both likelihood and prior information in their estimates. This was calculated overall, for each condition, and for each block.

#### Bayesian optimal sensory weights

If participants perform according to the Bayesian optimum as portrayed in Eq. (1), then the optimal values for the slopes/sensory weights should be equal to the perceived $\frac{\sigma_P^2}{\sigma_L^2+{\sigma}_P^2}$, where ${\sigma}_P^2$ is the variance associated with the prior (narrow prior ${\sigma}_P^2={0.04}^2$; wide prior ${\sigma}_P^2={0.136}^2$) and ${\sigma}_L^2$ is the variance associated with the likelihood (in this instance, narrow likelihood ${\sigma}_L^2=\frac{0.096^2}{5}$; wide likelihood ${\sigma}_L^2=\frac{0.24^2}{5}$). These calculations of Bayesian optimality refer to posterior computations, integrating the relative uncertainty of both prior and likelihood information.

#### Trial-by-trial sensory weight

Equation (1) can be rewritten to calculate an instantaneous sensory weight as an indicator of participants reliance on likelihood relative to prior information on any given trial:

3 $${sw}_{trial}=\frac{X_{est}-{\mu}_P}{\ {\mu}_L-{\mu}_P}$$

where *X*_*est*_ is the participants estimated position of the coin on a given trial (net location), *μ*_*P*_ is the mean of the mean of the prior (assumed at the center of the screen), and *μ*_*L*_ is the mean of the likelihood (the center of the five blue dots for that trial). To ensure the trial-by-trial sensory weight varied from 0 to 1, we used a log-transformation for analyses.

#### Subjective likelihood variance

The likelihood-only task can be used to determine a proxy for participants subjective likelihood variance or sensory noise (Randeniya et al., 2021). This is determined by the variance of the participants estimates of the mean (*μ*_*est*_) relative to the true mean of the splashes (*μ*_*L*_):

4 $${\sigma}_{LS}^2=\frac{\Sigma {\left({\mu}_{est}-{\mu}_L\right)}^2}{nTrials}$$

where the number of trials (nTrials) was equal to 100 in the likelihood-only task.

#### Subjective prior variance

To estimate participants subjective model of where each thrower would throw the coin (subjective prior variance, ${\sigma}_P^2$), the sensory weight from Eq. (2) can be rearranged as follows:

5 $${\sigma}_P^2=\frac{\sigma_L^2\ast sw}{\left(1- sw\right)}$$

In this equation, ${\sigma}_L^2$ can be assumed to be the objective likelihood variance (i.e., the variance of the splashes by design), or alternatively, this can be estimated from participants subjective likelihood variance, ${\sigma}_{SL}^2$ (as calculated in Eq. 4).

### Statistical analysis

Firstly, mean estimation error was used as a criterion to detect poor performance or low effort, with four participants excluded in the likelihood only task, and two participants excluded in the main task (z-score greater than 3). To determine differences in key parameters across the two timepoints, *t* test and ANOVAs were calculated. A log-transformation was applied to non-parametric data to normalize the distribution. Test–retest reliability was then analyzed with Pearson correlations and intraclass correlations, ICC (2, 1), using a two-way random effects model based on absolute agreement (Koo & Li, 2016). The general formula for the traditional ICC can be calculated as follows:

6 $$p=\frac{\sigma_0^2}{\sigma_0^2+{\sigma}_{\varepsilon}^2}$$

where ${\sigma}_0^2$ refers to between-person variance and ${\sigma}_{\varepsilon}^2$ refers to within-person variance. ICCs with values of < 0.5 were interpreted as poor, 0.5–0.75 as fair, 0.75–0.90 as good, and > 0.90 as excellent reliability (as suggested by Koo & Li, 2016). Additionally, split-half reliability was analyzed for trial-by-trial estimates of estimation error and sensory weight at each timepoint separately. This was done using a permutation-based split-half approach (Parsons, 2021), in which data is repeatedly split into two halves and the reliability estimate is calculated for each split, then averaged to provide a more stable estimate of reliability. We used 5000 random splits as recommended by Parsons (2021) with Spearman–Brown corrected estimates and their 95% percentile intervals reported as a measure of internal consistency. Finally, exploratory analyses of trial-by-trial sensory weight included implementing a hierarchical model that allows for individually varying intraclass correlations, to address cross-trial variability and directly test for homogenous within-person variance in test–retest reliability (Williams et al., 2022; using vICC package in R), which can be calculated as:

7 $${p}_i=\frac{\sigma_0^2}{\sigma_0^2+\exp \Big[{\eta}_0+{\mu}_{1i}\Big]}$$

where *i* refers to the *i*th individual, ${\sigma}_0^2$ refers to between-person variance,*η*_0_ represents the average of individual variances, and *μ*_1*i*_ represents individual departures from the fixed group effect. This model computes person-specific ICC, allowing us to determine which (and how many) individuals belong to the common variance ICC model (as described in Eq. 6). This means that trial-by-trial sensory weight data across both testing sessions is used to inform an individual ICC metric which is unique to each participant. Like the traditional ICC approach, higher individual ICC estimates demonstrate more stability or similar scores across testing sessions, while lower individual ICC estimates demonstrate larger differences across testing sessions. If *most* participants demonstrate high individual ICC estimates, we can be more confident that this reflects *true stability* in performance across testing sessions (and vice versa for low individual ICC estimates). In other words, we are more interested in whether the majority of participants demonstrate similar individual ICC estimates, or whether the sample has a large degree of variation in individual ICC scores. Given the novelty of this methodological approach, there are no clear guidelines for the proportion of participants demonstrating low individual ICC scores to constitute *true change*. All visualizations and analyses were performed in R (version 2022.02.2).

## Results

### Participants

Data from a total of 37 participants was collected from participants that completed both testing sessions, with demographic information provided in Table 1. Demographic information of participants that only completed timepoint 1 and not timepoint 2 (i.e., dropped out of the study) can be found in the Supplementary (Table S1).

**Table 1:** Demographic information collected from participants (*n* = 37) that completed the experiment

Age (years)	M	SD	Range
	22.62	6.72	19–57
Gender	Female	Male	Other
	29	6	2
Highest level of education	Primary school	Secondary school	Tertiary education
	0	26	11
English as a first language	Yes	No	
	17	20	
Handedness	Left-handed	Right-handed	No preference
	5	31	1

### Performance accuracy

An analysis of performance accuracy in the likelihood-only task revealed no significant difference in overall mean estimation errors between timepoint 1 and timepoint 2 (*t(*65) = 1.8, *p* = .076, 95% CI [– 0.011, 0.0005]). When comparing performance across each condition, we found significantly greater mean estimation errors in the wide likelihood condition compared to the narrow likelihood condition at timepoint 1 (*t*(32) = 5.48, *p* = 5.26 × 10^-8^, 95% CI [0.0167, 0.009]) and timepoint 2 (*t*(32) = 5.49, *p* = 4.83 × 10^-6^, 95% CI [0.021, 0.009]). This is intuitive, given that the wide likelihood condition provides less certain information about the coin’s location, replicating previous findings (Goodwin et al., 2022). When considering performance accuracy in the main task, there was no significant difference in mean estimation error between timepoint 1 and timepoint 2 (*t*(139) = – 0.73, *p* = 0.467, 95% CI [– 0.0086, 0.004]). When comparing performance across each condition, a two-way ANOVA revealed a main effect of prior (Pw>Pn; Timpoint 1: *F* = 67.62, *p* = 1.41 × 10^–13^; timepoint 2: *F* = 30.63, *p* = 1.55 × 10^-7^) and a main effect of likelihood (Lw>Ln; timepoint 1: *F* = 109.26, *p* < 2 × 10^–16^; timepoint 2: *F* = 69.10, *p* = 8.54 × 10^–14^) across both timepoints. This indicates that more estimation errors were occurring in the conditions with greater uncertainty, as expected by the task design.

We estimated the internal consistency of overall estimation error in the main task using a permutation-based split-half approach (Parsons, 2021) with 5000 random splits. The Spearman–Brown corrected split half internal consistency measure was shown to demonstrate good to excellent reliability at timepoint 1 (*r*_SB_ = 0.82, 95% HDI [0.72, 0.90]) and good to moderate reliability at timepoint 2 (*r*_SB_ = 0.76, 95% HDI [0.57, 0.88]), based on recommendations from Koo and Li (2016). Additionally, intraclass correlation coefficient of overall estimation error demonstrated moderate test–retest reliability (ICC(2, 1) = 0.47, 95% CI [0.16, 0.68]) with a positive correlation between testing sessions (*r* = 0.67, *p* = 1.83 × 10^–5^, bootstrapped 95% CI [0.49, 0.88]). This indicates that performance accuracy was stable within testing sessions, as well as across the two timepoints.

### Average sensory weights suggests that participants perform in a Bayesian manner (non-optimally)

**Fig. 2:**
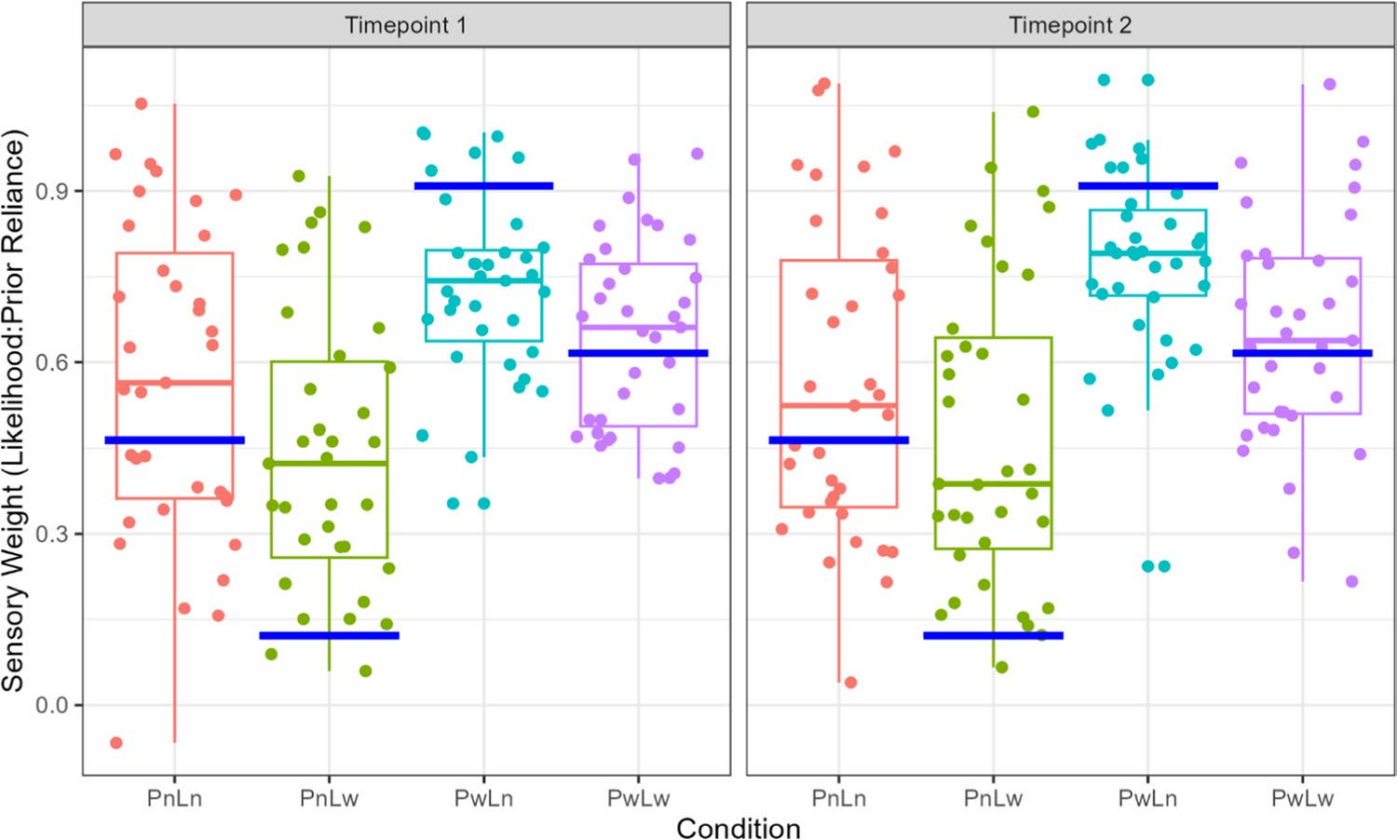
Comparison of sensory weights across each condition at timepoint 1 and timepoint 2. *Note.* Sensory weight for each participant is calculated by the slope of the regression between the true center of the likelihood and participant’s estimates of the coin’s location for each condition. A sensory weight closer to 1 indicates greater reliance on likelihood, whilst a sensory weight closer to 0 indicates greater reliance on prior. *Blue lines* indicate the Bayesian optimal computation of the coin’s location, based on the posterior integration of uncertainty in both prior and likelihood information. Conditions: PnLn = narrow prior, narrow likelihood *(red dots*); PnLw = narrow prior, wide likelihood (*green dots*); PwLn = wide prior, narrow likelihood (*teal dots*); PwLw = wide prior, wide likelihood (*purple dots*).

Sensory weight (likelihood to prior reliance) was calculated overall and for each condition across both timepoints. We found no significant difference between the average sensory weight at timepoint 1 (*M* = 0.63, *SD* = 0.17) and timepoint 2 (*M* = 0.65, *SD* = 0.18; *t*(34) = – 1.15, *p* = .259). Analysis of sensory weights across conditions, showed a main effect of prior across both timepoints (Pw>Pn; timepoint 1: *F* = 25.29, *p* = 1.53 × 10^-6^, timepoint 2: F =24.70, *p* =1.98 × 10^-6^), as well as a main effect of likelihood (Ln>Lw; timepoint 1: *F* = 8.46, *p* = .0042, timepoint 2: *F* = 7.94, *p* = .0056). This indicates that participants relied more on likelihood information when the prior was more uncertain but relied less on likelihood information when the likelihood was more variable, as expected. Despite this, Wilcox ranked tests showed that median sensory weights across three of the four conditions were significantly different from Bayesian optimal scores at each timepoint (excepting PwLw condition; see supplementary S2). Although participants generally deviated from Bayesian optimal, their patterns of performance were verging towards optimality across each condition, as shown in Fig. 2.

### Sensory weight parameter has both good internal stability and test–retest reliability

**Fig. 3:**
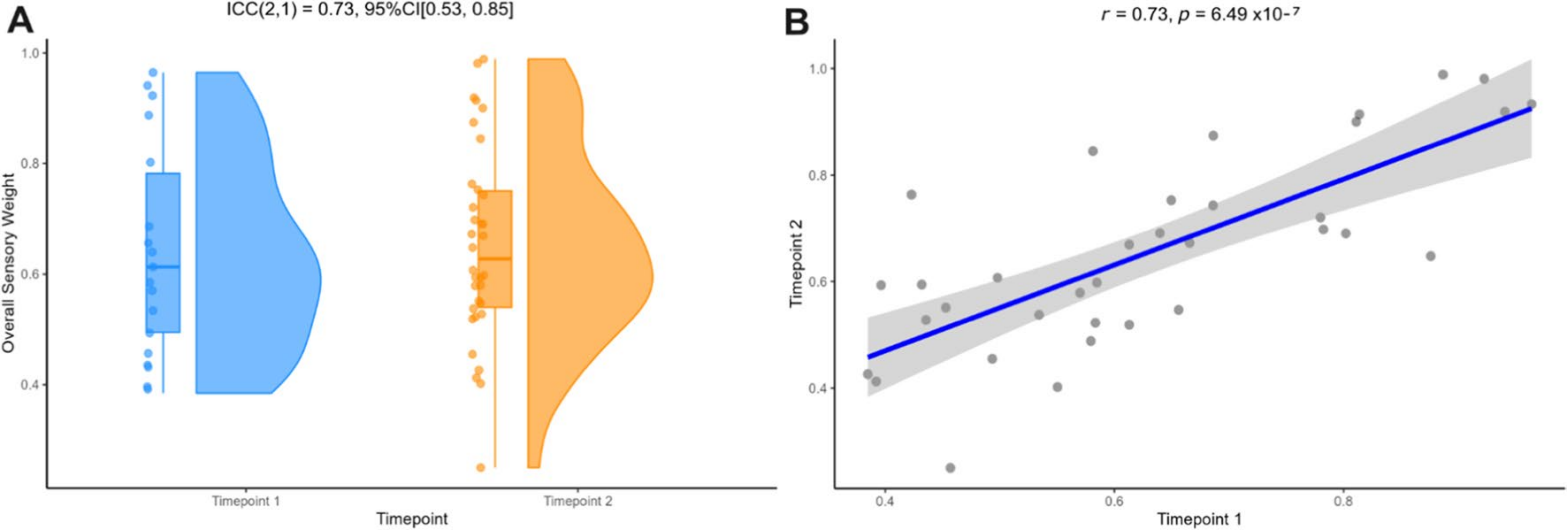
Average overall sensory weight scores demonstrated **A** high test–retest reliability across two timepoints and **B** a strong positive Pearson correlation between timepoint 1 and timepoint 2

Trial-by-trial sensory weight (see Eq. (3) for calculation) demonstrated good-to-excellent internal consistency at timepoint 1 (r_SB_ = 0.87, 95% HDI = [0.80, 0.93]) and at timepoint 2 (r_SB_ = 0.88, 95% HDI = [0.81, 0.93]), as measured with a Spearman–Brown corrected split-half measure with the permutation approach. To evaluate whether individuals’ average sensory weight (see Eq. 2) remained stable across the two timepoints, we performed a test–retest analysis using Pearson correlation across sessions. Further, we calculated the intraclass correlation coefficient (ICC2, 1) which reflects the absolute agreement between measurements. Overall sensory weight showed good test–retest reliability (ICC(2, 1) = 0.73, 95% CI [0.53, 0.85]) with a strong positive Pearson correlation between timepoint 1 and timepoint 2 (*r* = 0.73, *p* = 6.49 × 10^–7^, bootstrapped 95% CI [0.59, 0.87]) as shown in Fig. 3. When considering individuals’ sensory weights across conditions, split-half reliability analyses demonstrated good to moderate internal consistency at both timepoints (supplementary S3). Similarly, measures of intraclass correlation coefficients demonstrated good to moderate test–retest reliability across the two testing sessions (supplementary S3).

### Subjective likelihood uncertainty (likelihood-only task) showed weak test–retest reliability between timepoints 1 and 2

**Fig. 4:**
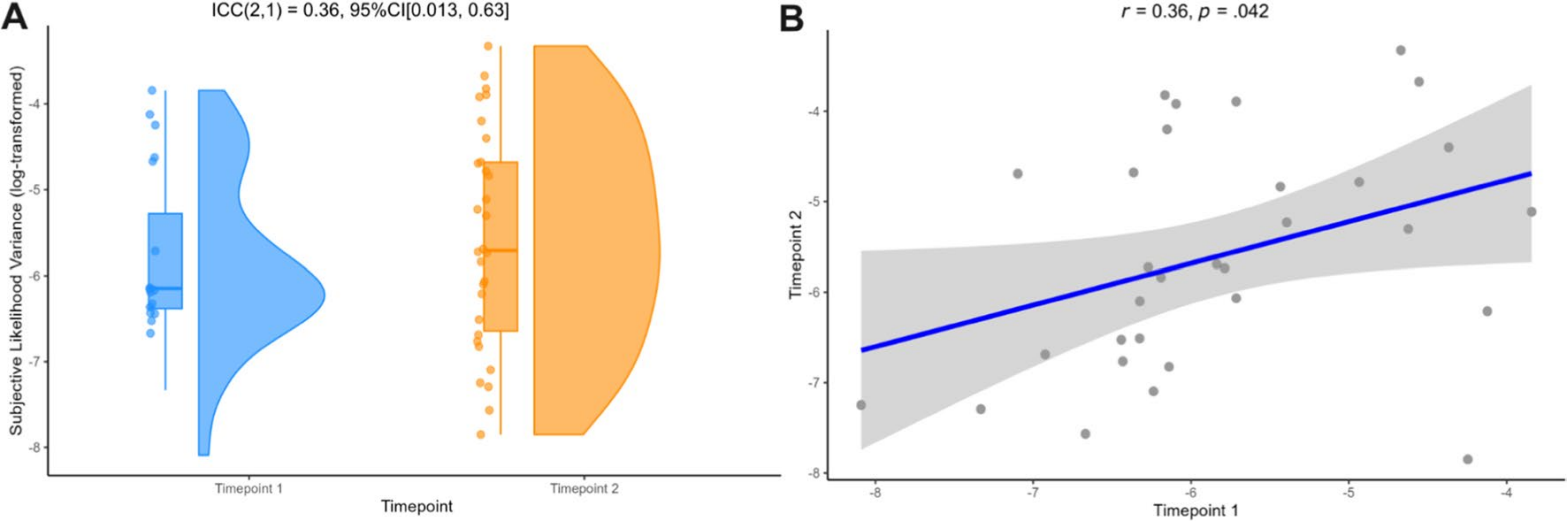
Average subjective likelihood variance scores from the likelihood-only task demonstrated **A** weak test–retest reliability across two timepoints and **B** a positive correlation between timepoint 1 and timepoint 2

Subjective likelihood variance was calculated in the likelihood-only task as a proxy of how much participants perceived uncertainty in likelihood information (i.e., distribution of the five blue dots) to vary across the task. Unexpectedly, there was no significant difference between average scores of subjective likelihood variance in the narrow condition, compared to the wide condition at both timepoint 1 (*t*(30) = 0.165, *p* = .869) and timepoint 2 (*t*(30) = 1.99, *p* = .066). Overall subjective likelihood variance scores were calculated for each participant, showing no significant difference between timepoint 1 (*M* = 0.0048) and timepoint 2 (*M* = 0.0074, *t*(30) = 1.42, *p* = .166). Before reliability analyses were conducted, log-transformations were applied to subjective likelihood variance scores to normalize the distribution of data (see Supplementary S4). Following this, ICC analysis of overall subjective likelihood variance scores demonstrated weak test–retest reliability between timepoint 1 and timepoint 2 (ICC(2, 1) = 0.36, 95%CI [0.013, 0.63]), with a positive correlation between timepoints (*r* = 0.36, *p* = .042, bootstrapped 95%CI [0.0079, 0.74]; see Fig. 4).

### Subjective prior uncertainty showed good to moderate test–retest reliability between timepoints 1 and 2

**Fig. 5:**
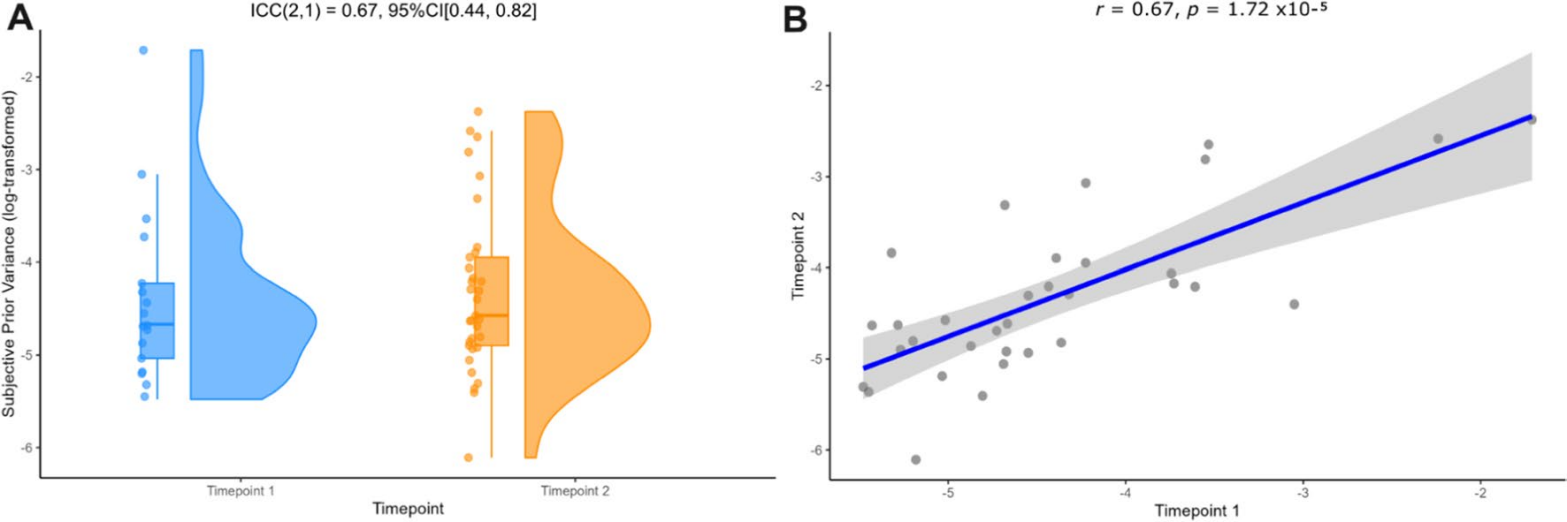
Average subjective prior variance scores demonstrated **A** high test–retest reliability across two timepoints and **B** a positive Pearson correlation between timepoints 1 and 2

Considering the lack of difference in subjective likelihood variance observed between narrow likelihood and wide likelihood conditions (from the likelihood-only task), the objective likelihood variance was instead used to calculate subjective prior variance (see Eq. 5). This, along with overall sensory weights, were utilized as a proxy to determine how much participants were perceiving uncertainty in the prior information (i.e., accuracy of thrower) to vary across the task. A comparison of subjective prior variance across conditions revealed no main effect of prior at timepoint 1 (*F* = 3.45, *p* = .066), but a main effect of prior at timepoint 2 (Pw>Pn; *F* = 5.33, *p* = .023). This shows that participants were more likely to perceive uncertainty in prior information when it was objectively more uncertain (i.e., in the wide prior condition). However, we also unexpectedly found a main effect of likelihood (Lw>Ln; timepoint 1: *F* = 13.17, *p* = 4.31 × 10^–4^; timepoint 2: *F* = 13.13, *p* = 4.34 × 10^–4^), suggesting that the likelihood condition was also influencing perceived uncertainty in prior information. Overall subjective prior variance was also calculated for each participant, in which we found no significant difference between timepoint 1 (M = 0.018) and timepoint 2 (M = 0.020, *t*(32) = – 0.30, *p* = .77). Data were normalized with a log-transformation before reliability analyses were conducted (see Supplementary S5). We found high test–retest reliability (ICC(2, 1) = 0.67, 95% CI [0.44, 0.82]), with a positive Pearson correlation between timepoint 1 and timepoint 2 (*r* = 0.67, *p* = 1.72 × 10^-5^, bootstrapped 95% CI [0.50, 0.86]; see Fig. 5).

### Exploratory analyses: Individually varying intra-class correlation coefficients

Traditional ICC analyses rely on mean point estimates with the assumption of a common within-person variance model, suggesting that each individual is adequately described by the average within-person variance. Rouder and Haaf (2019) demonstrated that average aggregates of subject-level data can greatly attenuate measures of reliability. Instead, they suggest that modeling individual-level variability in trial-by-trial parameters can yield robust individual differences. A novel hierarchical-modeling approach developed by Williams et al. (2022) can verify whether this average-level reliability is generalizable to the individual level. This approach tests for homogeneity of within-person variance with individually varying intraclass correlation coefficients. In other words, it allows identification of which, and how many individuals belong to a common variance model (i.e., which individuals the traditional ICC is representative of), and which individuals fall outside that common variance. This allows for the possibility of individual differences in test–retest reliability, renouncing the assumption that individuals are unlikely to deviate from the average. Whilst this technique provides an excellent opportunity to deeply characterize individual differences in this research, its novelty renders these analyses exploratory at this stage. For these analyses, we computed individually varying ICCs of trial-by-trial sensory weight estimates (see Eq. 3) separated by conditions representative of trial types. The models were fitted with the R package vICC as described in Williams et al. (2022).

**Fig. 6:**
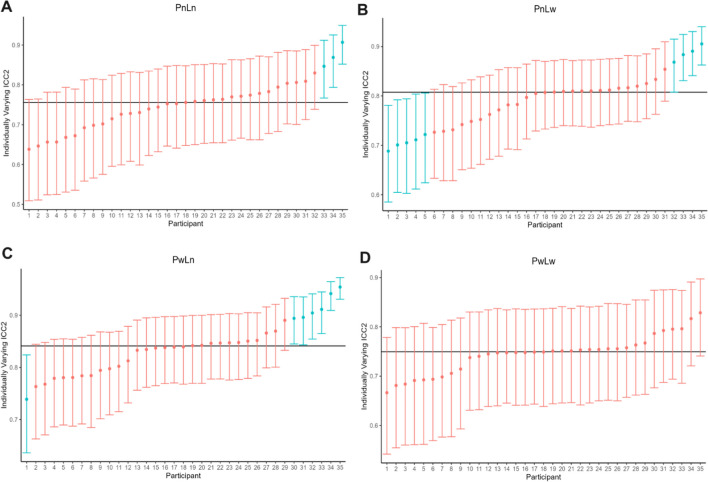
Varying intraclass correlation coefficients of trial-by-trial sensory weight across each condition. *Note.* A) narrow prior narrow likelihood (PnLn), B) narrow prior wide likelihood (PnLw), C) wide prior narrow likelihood (PwLn), and D) wide prior wide likelihood (PwLw). These plots show which individuals are homogenous within in the common variance model (in *red*), and which individuals fall outside the ‘traditional’ or average ICC (in *blue*). The traditional mean point estimate ICC is demonstrated as the *black horizontal line* in each plot, while individually varying ICCs are demonstrated as a point estimate and confidence interval around that estimate for each participant

Across each condition, we found that individually varying ICC2 estimates of most participants belonged to the common variance model, as shown in Fig. 6. The wide prior wide likelihood condition (PwLw) demonstrated the most homogeneity in test–retest estimates, whereby all participants belonged to the common variance model (as depicted in red). Whilst the narrow prior narrow likelihood condition (PnLn) demonstrated the most heterogeneity, only nine participants fell outside what was described by the common variance model (as depicted in blue). In other words, there was not a large degree of heterogeneity in individually varying ICC calculations across each condition, suggesting that the common variance model is an accurate descriptor of test–retest reliability across the majority of individuals in this sample. Additionally, these analyses show that confidence intervals around point estimates fall within a wide range of poor to excellent test–retest reliability depending on the individual, which demonstrates variable interpretability across the sample.

## Discussion

The aim of the present study was to determine whether individual differences in the precision weighting of prior to likelihood information remain temporally stable over two testing sessions. This was to empirically examine the assumption that this measure acts as a stable characteristic of Bayesian information integration. Precision weighting was measured with a behavioral paradigm that parameterizes the weighting afforded to prior and likelihood information via orthogonal manipulation of uncertainty across trials. Our data demonstrated high internal consistency and good test–retest reliability in individuals’ average sensory weight across the task, as well as good to moderate test–retest reliability in individuals’ average sensory weight across conditions. At the individual level, exploratory analyses investigating individually varying ICCs across conditions suggested high homogeneity with a common variance model, indicating good to moderate test–retest reliability of trial-by-trial sensory weights amongst individuals. Thus, sufficient temporal stability of this parameter at both the average and individual level demonstrates its suitability as a cognitive marker of individual differences in perceptual decision-making in the general population (Rouder & Haaf, 2019). This has important implications for the empirical utility of Bayesian belief updating in future clinical applications of this task (Parsons et al., 2019).

Importantly, the reliability of the precision weighting of prior to likelihood information in this study (ICC(2,1) = 0.73, 95% CI [0.53, 0.85]) is comparable to similar performance-based indicators of perceptual inference in other task designs. For example, a recent study investigated the influence of cross-modal auditory priors in the perception of bistable visual stimuli, revealing high temporal stability in individual differences of perceptual inference (median ICC = 0.83, 95% CI [0.61, 0.95]; Pálffy et al., 2021). Similarly, behavioral measures of cognitive flexibility in a probabilistic reversal learning task were also found to have excellent reliability (derived from mixed-effects models over two testing sessions; Waltmann et al., 2022). This provides evidence for the generalizability of reliable individual differences in behavioral measures of perceptual inference in healthy populations. While test-retest reliability is important to ensure a parameter acts as a stable characteristic, it is also important to verify the internal reliability of a parameter, to ensure the construct does not rapidly fluctuate within individuals. Internal consistency estimates such as split half reliability can provide an upper bound on test–retest reliability (Karvelis et al., 2023). In the current study, the internal consistency of sensory weight was found to be good to excellent at timepoint 1 (r_SB_ = 0.87, 95% HDI = [0.80, 0.93]) and at timepoint 2 (r_SB_ = 0.88, 95% HDI = [0.81, 0.93]), meaning that individuals’ precision weighting of prior to likelihood information has high reliability within the task itself.

Task-based measures of perceptual inference are often used as trait-like characteristics to assess the relationship with clinical symptoms across a spectrum of disorders such as schizophrenia (Deserno et al., 2020; Schlagenhauf et al., 2014; Weilnhammer et al., 2020) and autism spectrum disorder (Kreis et al., 2021; Randeniya et al., 2021), as well as their non-clinical trait-like correlates (Goodwin et al., 2022; Kreis et al., 2023). Mounting evidence for the reliability of distinct perceptual phenotypes across task-based measures of cognition support an important development in the translation of computational measures to clinical practice, particularly as a diagnostic tool in psychiatric research (Gibbs-Dean et al., 2023; van Leeuwen et al., 2021). For this translation to be effective, the reliability of such measures of perceptual inference should also be established in clinical populations before empirical use. The utility of computational psychiatry is growing, not only as a tool to investigate mechanisms underlying cognition and behavior, but also to inform techniques in therapy (Pott & Schilbach, 2022), predict treatment response (Hauke et al., 2023), predict transitions from ‘at risk’ states (Hauke et al., 2023), and the potential retraining of perceptual priors in clinical disorders (Lyndon & Corlett, 2020). This demonstrates the importance of generating robust and reliable tools to formally assess beliefs, how they change over time, and how they relate to observable behavior.

Furthermore, although sufficient temporal stability of the precision weighting of prior to likelihood information was demonstrated, the subjective uncertainty of likelihood information was found to have poor test–retest reliability in our task. Practice effects could hinder the reliability of this parameter, as exposure to the likelihood-only task (from which this parameter is calculated) in the second testing session is no longer naïve (Randeniya et al., 2021). In the first testing session, the coin task is initially presented without manipulating prior information (i.e., the likelihood-only task), in order to yield a proxy for participants’ unbiased estimates of the subjective uncertainty associated with likelihood information. In the second testing session, the likelihood-only task is again presented before the main task, however participants’ previous experience with the main task might be biasing their responses, due to a carryover of prior information from the first testing session to the second. Thus, following up this study with further testing sessions would provide insight into whether practice effects impact the strategy being used (indicative of individuals’ subjective likelihood uncertainty) in the likelihood-only task. Alternatively, increasing the timeframe between testing sessions might provide further insight into the trade-off between practice effects and stability in responding (Karvelis et al., 2023; Zech et al., 2022).

In sum, our findings provide support for temporal stability within individuals in the precision weighting of likelihood to prior information in single task-based measures. However, it is unclear whether these findings are generalizable to other paradigms that tap into processes of Bayesian information integration. Research in this area so far has yielded contradictory findings. In attempts to understand state vs trait alterations in predictive processing, previous research has utilized different methods of prior induction to determine whether a single factor could explain performance (such as a relative reliance on priors) across multiple tasks (Andermane et al., 2020; Koblinger et al., 2021). For example, Tulver et al. (2019) investigated whether the tendency to rely on priors across multiple paradigms with noisy or ambiguous perceptual input could co-explain performance, and whether this was linked to autistic or schizotypal traits in non-clinical populations. Surprisingly, they found no single factor to explain individual differences or a common reliance on priors across four tasks with visual illusions. This might suggest that different methods of prior induction may recruit distinct neural mechanisms that operate at different levels of the visual hierarchy. Similarly, Andermane et al. (2020) investigated whether a general tendency to see the expected is general, or method specific with different facilitatory effects of perceptual priors. While they found that individual differences in expectation-based biases are closely related to attentional ability, test–retest reliability is required to decisively measure whether this operates as a consistent phenotypic difference. Whilst these computational cognitive models are often context-specific, it is yet to be verified whether measures of perceptual inference in the coin task can also predict performance across other, similar tasks.

One limitation of our task design was that the order in which participants observed narrow versus wide uncertainty in prior information was not counter balanced across testing sessions. Ensuring that each participant received a different version of the task across the two testing days could potentially improve expectation-based biases in responding and further limit practice-effects. Additionally, to ensure robust replicability of the online-version of this task, future research should verify the test–retest reliability across online and in-person testing sessions, to ensure the consistent performance of psychometrics (Zech et al., 2022). As a quality control measure for our online testing, participants with particularly high mean estimation error scores were removed, as this was deemed to be an indicator of poor adherence or engagement with the task. Additionally, participants were asked to complete practice tasks prior to completion of the main task, to ensure that they understood what was required in the main task. While online testing has many resourceful benefits, an advantage of in-person cognitive-behavioral testing is that extraneous environmental factors can be more stringently controlled. Although cognitive processes will inevitably fluctuate across testing sessions, controlling for the time of day (i.e., circadian rhythm; Bedder et al., 2023) and assessing mood-related fluctuations (Eldar et al., 2018) could provide insight into state-related differences across testing sessions that might impact measurement reliability. Future research should include more sensitive investigations of state-like fluctuations, as these could provide insight into deviations from homogenous common variance models which may in fact be clinically meaningful (Karvelis et al., 2023; Sullivan-Toole et al., 2022). Despite this, testing perceptual inference in clinical settings might also lack this stringent stability and have inevitable fluctuations, suggesting that our research might hold better generalizability to clinical testing than a strictly controlled lab-based setting. To further aid generalizability, these findings should be replicated in a more age and demographically diverse sample, to ensure translational value to clinical populations. Similarly, the small sample size limits the generalizability of these findings, meaning that the results should be considered a promising starting point for the reliability of sensory weighting, with future replications in a larger sample to provide more conclusive evidence.

This study demonstrated good internal consistency and sufficient average and individual estimates of test–retest reliability in the precision weighting of prior to likelihood information in a perceptual decision-making task. This provides evidence that individual differences in task-based measures of Bayesian information integration perform as a stable characteristic. These results support the characterization of a latent computational measure as a means to capture potentially clinically relevant individual differences in computational psychiatry (Karvelis et al., 2023). This verification of psychometrics of cognitive-behavioral tasks should be standard practice before exploring their relationship with symptoms in clinical disorders and sub-clinical trait-like correlates in the general population.

### Supplementary information

ESM 1 (DOCX 464 kb)
