# Supplementary material for: Temporal stability of Bayesian belief updating in perceptual decision-making
Source: Behav Res Methods. 2023 Dec 21;56(6):6349–62. doi: 10.3758/s13428-023-02306-y (PMC11335944; doi:10.3758/s13428-023-02306-y)
Supplement: Supplementary file 1 — (DOCX 464 kb) [file 13428_2023_2306_MOESM1_ESM.docx]

**Supplementary**

**Table S1**

*Demographic information of participants (n=28) that only completed timepoint 1 and not timepoint 2 (i.e., dropped out of the study)*

| **Age (years)** | **M** | **SD** | **Range** |
| --- | --- | --- | --- |
|  | 20.64 | 1.48 | 19 - 26 |
| **Gender** | **Female** | **Male** | **Other** |
|  | 21 | 7 | 0 |
| **Highest level of education** | **Primary school** | **Secondary**  **school** | **Tertiary education** |
|  | 0 | 22 | 6 |
| **English as a first language** | **Yes** | **No** |  |
|  | 17 | 11 |  |
| **Handedness** | **Left-handed** | **Right-handed** | **No preference** |
|  | 3 | 24 | 1 |

**Table S2** *Median Sensory Weights Significantly Different from Bayesian Optimal Sensory Weights in Three of the Four Conditions across Both Timepoints*

|  | **Timepoint 1** | | | **Timepoint 2** | | |
| --- | --- | --- | --- | --- | --- | --- |
|  | Median | Optimal | p-value | Median | Optimal | p-value |
| PnLn | 0.571 | 0.465 | 0.044 | 0.561 | 0.465 | 0.078 |
| PnLw | 0.431 | 0.122 | <.001 | 0.467 | 0.122 | <.001 |
| PwLn | 0.734 | 0.909 | <.001 | 0.785 | 0.909 | <.001 |
| PwLw | 0.645 | 0.616 | 0.351 | 0.648 | 0.616 | 0.395 |

**Table S3***Internal Consistency and Test Re-Test Reliability of Sensory Weight across Each Condition*

|  | **Split-half reliability** | | | | **Test re-test reliability** | | | |
| --- | --- | --- | --- | --- | --- | --- | --- | --- |
|  | Timepoint 1 | | Timepoint 2 | |  | | | |
| **Condition** | ***r_S_*_B_** | 95%CI | ***r_S_*_B_** | 95%CI | **ICC(2,1)** | 95%CI | **r** | 95%CI |
| PnLn | 0.67 | [0.49, 0.82] | 0.52 | [0.25, 0.73] | 0.62 | [0.37, 0.79] | 0.60 | [0.36, 0.85] |
| PnLw | 0.73 | [0.58, 0.85] | 0.69 | [0.50, 0.82] | 0.67 | [0.45, 0.82] | 0.66 | [0.40, 0.93] |
| PwLn | 0.67 | [0.49, 0.82] | 0.78 | [0.65, 0.88] | 0.41 | [0.11, 0.65] | 0.48 | [0.19, 0.79] |
| PwLw | 0.55 | [0.33, 0.74] | 0.69 | [0.52, 0.83] | 0.63 | [0.38, 0.79] | 0.62 | [0.44, 0.85] |

*Note.* Internal consistency of trial-by-trial sensory weights was measured with a spearman-Brown correction, split-half permutation approach. Split-half reliability was calculated for each condition, at each timepoint, along with a 95% confidence interval. Test re-test reliability was calculated for average sensory weights at each condition, using ICC2 which reflects absolute agreement between measurements, as well as the 95% confidence interval for this measure. Spearman-ranked correlation of individuals’ average sensory weight between timepoint 1 and 2 are also shown in the final column. Condition labels are as in Figure 2.

**Figure S4**

*
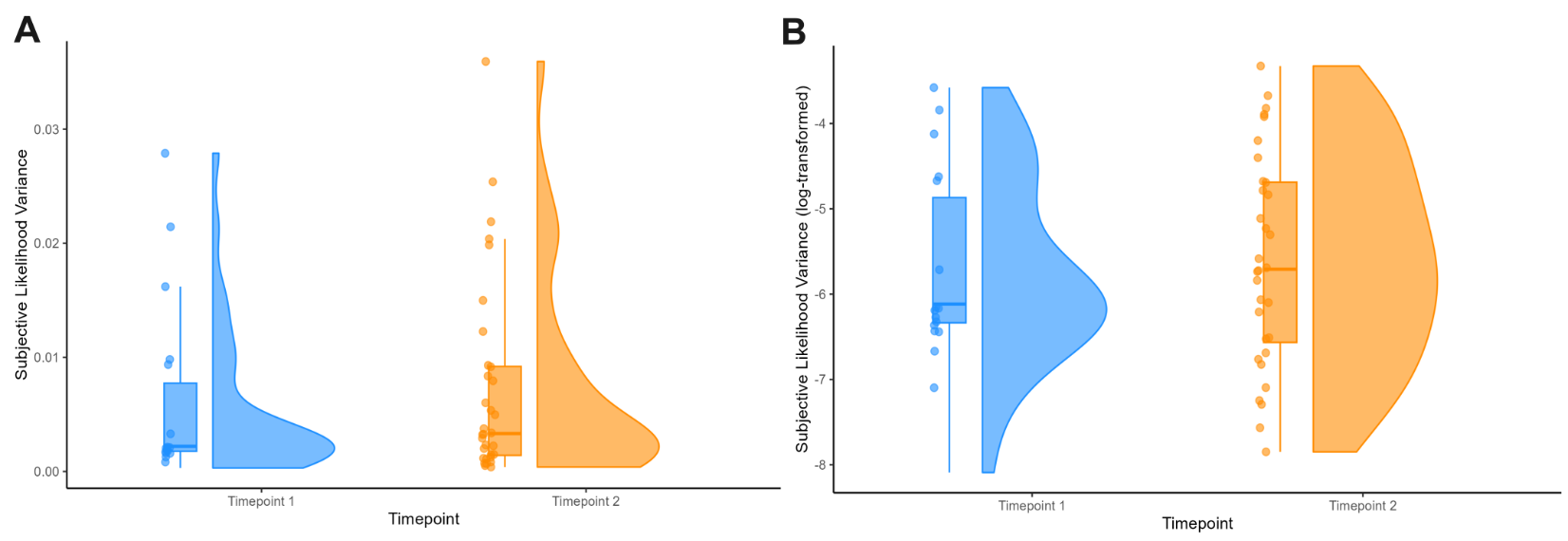
Comparison of A) Raw and B) Log-Transformed Distributions of Subjective Likelihood Variance Scores*

**Figure S5**

*
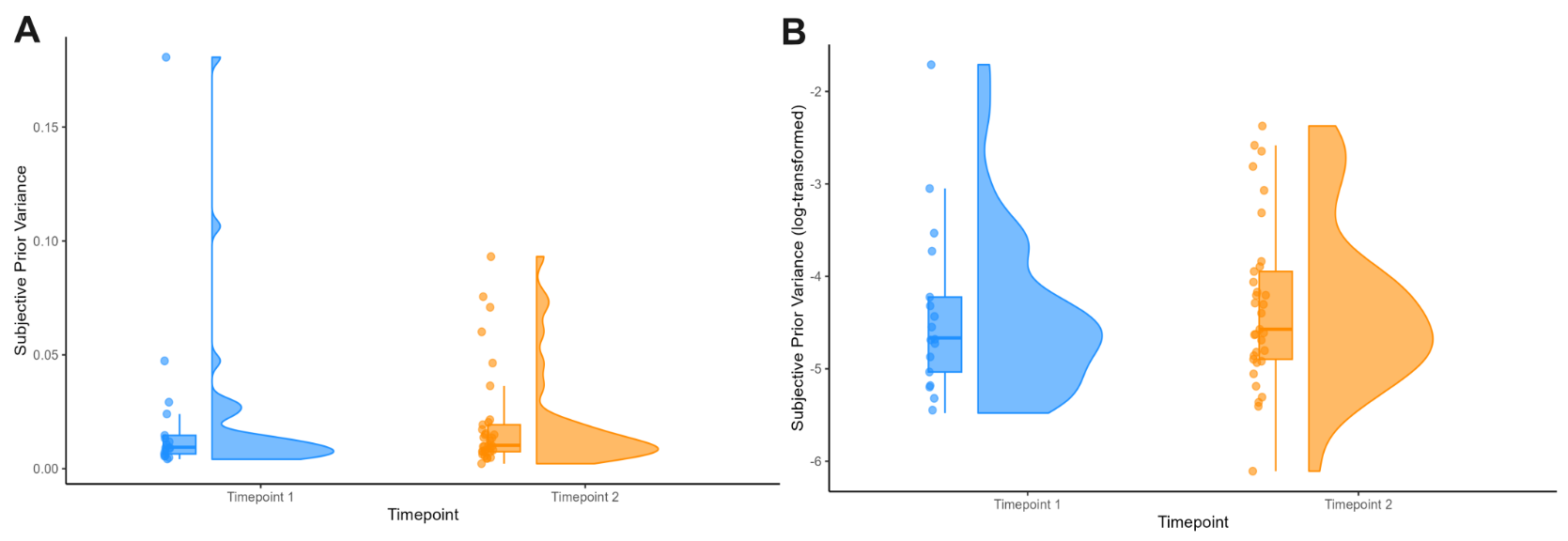
Comparison of A) Raw and B) Log-Transformed Distributions of Subjective Prior Variance Scores*
